# Overcoming resistance to immune checkpoint inhibitor therapy in acute myeloid leukemia

**DOI:** 10.3389/fonc.2026.1608433

**Published:** 2026-02-24

**Authors:** Vaibhav Agrawal, Amandeep Salhotra

**Affiliations:** Department of Hematology and Hematopoietic Cell Transplantation, Gehr Family Center for Leukemia Research, City of Hope National Medical Center, Duarte, CA, United States

**Keywords:** AML, checkpoint inhibitor, immune evasion, immune microenvironment, resistance

## Abstract

Acute myeloid leukemia (AML) is characterized by molecular, immunological, and clinical heterogeneity and this has been reflected with changing paradigms in the treatment landscape of AML in recent years. Despite high remission rates with conventional chemotherapy regimens, most patients eventually relapse and have disappointing outcomes in the relapsed setting, with estimated 5-year overall survival rates ranging from 10-15%. Immune checkpoint inhibitors (ICIs) restrain T-cell suppressive signals delivered through cytotoxic T-lymphocyte-associated protein 4 (CTLA-4) or programmed cell death protein 1 (PD-1)/programmed death-ligand 1 (PD-L1) pathways and promote antitumor immune responses and these agents have been explored successfully in the treatment of many malignancies. Although ICIs have demonstrated long-term clinical benefit in solid tumors such as melanoma or lung cancer, AML has historically been considered an immunologically “cold” tumor and responses have been less robust. Furthermore, the impact of T-cell functional states and the tumor microenvironment in response to standard chemotherapy regimens, molecularly targeted therapies, and immunotherapies is poorly understood. Recent advances in multimodal omics technologies have deepened our understanding of the dynamic cellular interactions at the AML immune interface and immune gene expression profiling can potentially be integrated with clinically validated molecular prognosticators to further stratify AML into potential tumor microenvironment subgroups that could more aptly respond to ICIICIs to reinvigorate dysfunctional T cells and modulate cytotoxicity. This review will attempt to summarize recent discoveries on T-cell functional states in AML and their impact on response to ICIICIs as well as acquired mechanisms of resistance to ICIICIs. Furthermore, strategies to potentially overcome resistance to ICIICIs in the immune-based treatment of AML will be highlighted to provide an outlook for future strategies.

## 1 Introduction

Acute myeloid Leukemia is a heterogeneous and aggressive malignancy characterized by the uncontrolled proliferation of immature myeloid cells in the bone marrow, leading to impaired hematopoiesis and life-threatening cytopenia. AML is known for its poor prognosis, with relapse and refractory disease being common, particularly among elderly patients. Despite recent improvements in the understanding of the molecular pathogenesis of AML, treatment outcomes, particularly in elderly or high-risk patients, remain suboptimal. Conventional chemotherapy regimens, including cytarabine-based induction, and allogeneic hematopoietic stem cell transplantation (HCT) are the standard of care for most patients, but relapse rates remain high. The immune system’s ability to identify and destroy malignant cells is a critical determinant of cancer progression, and immune evasion by tumor cells is a key challenge in the treatment of AML. Despite advances in chemotherapy and transplantation, the survival rates for AML patients unfortunately remain suboptimal. Over the past decade, immune checkpoint inhibitors) have revolutionized the treatment of various cancers, including solid tumors. However, their application in AML has been met with mixed success, prompting researchers to explore the mechanisms underlying resistance to checkpoint blockade therapy.

In recent years, ICIs have emerged as a novel and promising therapeutic class. These agents work by blocking inhibitory signals that dampen immune responses, thus enhancing T-cell activity against tumor cells. AML has multiple mechanisms to evade immune surveillance, including the expression of immune checkpoints like PD-L1, which interacts with PD-1 receptors on T-cells, and CTLA-4, which inhibits co-stimulation during T-cell activation. These checkpoints contribute to the suppression of anti-tumor immune responses and allow AML cells to escape immune destruction. CTLA-4 is another key checkpoint molecule that limits the activation of T-cells by interfering with co-stimulatory signals. It is upregulated in both AML cells and the tumor microenvironment, contributing to T-cell exhaustion and immune suppression. In this context, checkpoint inhibitors targeting PD-1/PD-L1, CTLA-4, and other immune checkpoints are being investigated in AML.

Checkpoint inhibitors, such as those targeting the programmed cell death-1 (PD-1)/programmed cell death-ligand 1 (PD-L1) pathway and cytotoxic T-lymphocyte antigen-4 (CTLA-4), work by blocking inhibitory signals that dampen T-cell responses, thereby enhancing anti-tumor immunity. In theory, AML is an ideal candidate for immune-based therapies, given its immune-modulatory properties and the growing recognition of the importance of the immune microenvironment in disease progression. Despite these promising theoretical foundations, clinical trials have demonstrated limited efficacy of ICIs in AML. Resistance to checkpoint blockade therapy has emerged as a significant obstacle to therapeutic success, and overcoming this resistance is crucial to unlocking the full potential of immunotherapy in AML treatment. Resistance to these therapies arises from various tumor-intrinsic, tumor microenvironment-mediated, and host-related factors. Addressing these resistance mechanisms is critical for enhancing the effectiveness of checkpoint blockade strategies in AML.

The purpose of this review is to provide a comprehensive analysis of the mechanisms underlying resistance to checkpoint inhibitors in AML. It will also explore strategies to overcome these barriers, focusing on combination therapies, modulation of the tumor microenvironment, and the use of novel immune-modulatory approaches.

## 2 Mechanisms of immune evasion in AML

**Figure 1:**
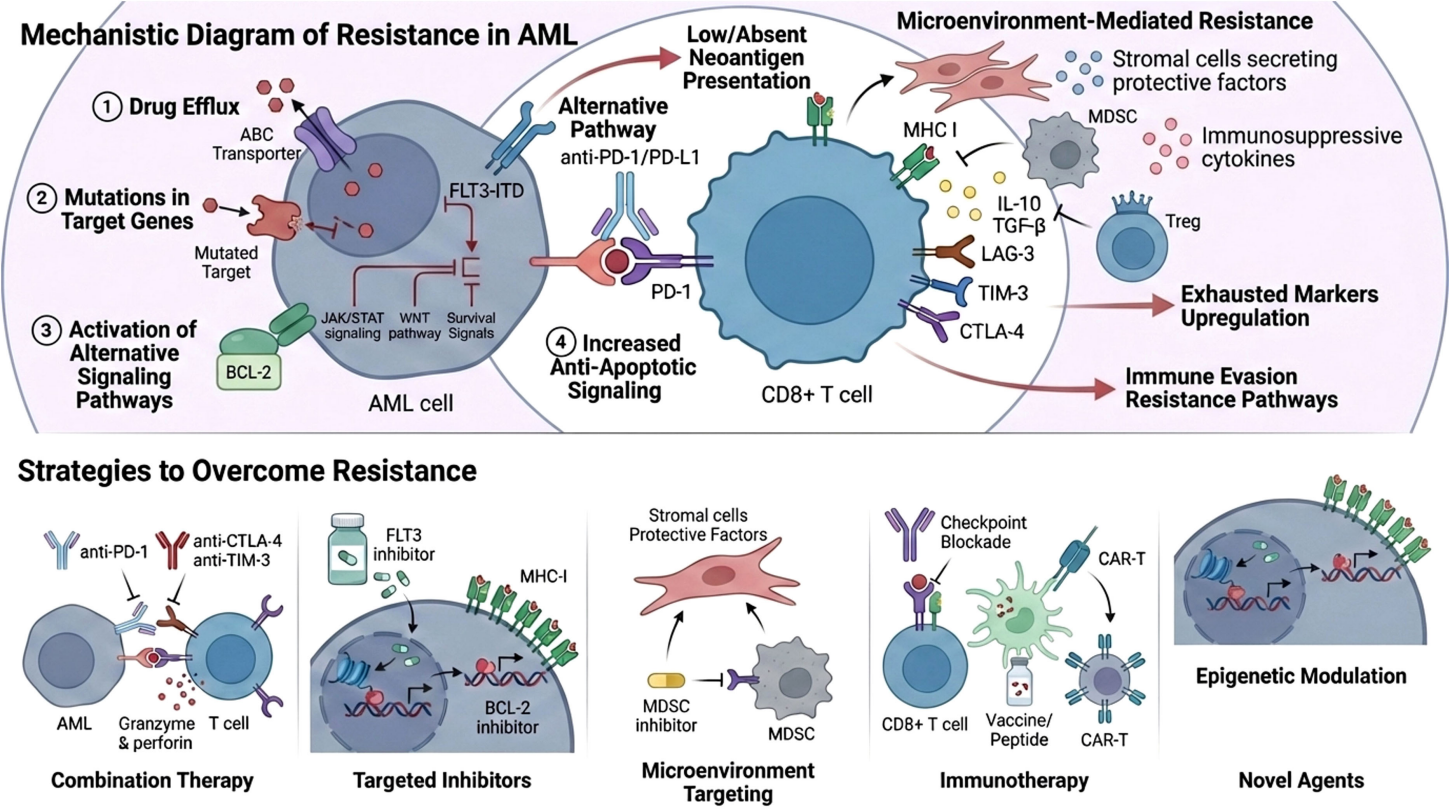
Schematic illustration outlining resistance patterns in AML as well as identified strategic approaches to overcome resistance.

The bone marrow microenvironment results from a complex network of intrinsic and extrinsic factors influenced by leukemic stem cells to induce immunosuppression and induce disease progression (**Figure 1**). The microenvironment changes are subsequently impacted by myeloid disease: in low-risk myelodysplastic syndrome (MDS), there is a noted prevalence of an inflammatory microenvironment with immunosenescence (1). In high-risk MDS and AML, the microenvironment is more influenced by clonal expansion and immunosuppression. In AML, the microenvironment affects all of the components of successful anti-tumor immune response, including low neo-antigen burden and defective antigen presentation, an imbalance between T-effector and regulatory T-cells (Tregs), T-cell exhaustion through the upregulation of immune checkpoint receptors and ligands, increased levels of myeloid suppressor cells (MDCs) suppressive macrophages, and overall T-cell activity (1–3).

### 2.1 Tumor-intrinsic factors

Tumor cells in AML exhibit several intrinsic resistance mechanisms that impair the efficacy of checkpoint inhibitors. The expression of PD-1/PD-L1 in AML blasts is significantly lower compared to solid tumors, reducing the effectiveness of PD-1/PD-L1 blockade (4). Additionally, AML cells often have a low mutational burden, limiting the generation of neoantigens required to elicit a strong T-cell response (5). The subsequent presence of fewer neoantigens results in reduced immunogenicity and a lower probability of effective T-cell mediated tumor clearance (6). Impairment of antigen presentation through mutations or downregulation of key molecules such as beta-2 microglobulin (B2M) can further hinder immune recognition by deficient antigen presentation and decreased recognition by cytotoxic T lymphocytes. Additionally, alterations in the MHC-II complex further impair the ability of antigen-presenting cells (APCs) to stimulate T-cell activation (7).

Furthermore, epigenetic modifications, including DNA methylation and histone alterations, contribute to immune evasion by suppressing the expression of immunogenic antigens and immune-activating molecules. DNA methyltransferases (DNMTs) and histone deacetylases (HDACs) can silence genes involved in antigen processing and presentation, limiting immune detection. AML has classically evaded immune recognition by promoter hypermethylation of tumor suppressor genes and immune-stimulatory molecules to reduce T-cell infiltration and blunt the immune response (8).

Other intrinsic resistance mechanisms include upregulation of alternative immune checkpoints such as T-cell immunoglobulin and mucin-domain containin-3 (TIM-3), lymphocyte activation gene-3 (LAG-3), and T-cell immunoreceptor with IgG and ITIM domains (TIGIT), which can bypass PD-1/PD-L1 blockade and sustain immune evasion (9). TIM-3 is highly expressed on AML blasts and leukemic stem cells and has been shown to play a critical role in suppressing anti-tumor immunity by effectively promoting T-cell exhaustion (10, 11). Similarly, upregulation of LAG-3 and TIGIT contribute to immune resistance by inhibiting T-cell activation and proliferation (12).

Additionally, dysregulation of key signaling pathways such as JAK/STAT, PI3K/AKT, and WNT/β-catenin can contribute to immune escape by altering cytokine signaling and downregulating antigen presentation machinery. In particular, JAK/STAT signaling has been implicated through hyperactivation of STAT3 and STAT5, which can promote an immunosuppressive environment through upregulating anti-inflammatory cytokines (13). AML cells frequently activate the PI3K/AKT/MTOR axis, which inhibits T-cell effector function and enhances immune evasion (14). AML blasts can inhibit NK/T cell function by releasing soluble forms of NKG2DL resulting in immune escape by this mechanism (15). Lastly, aberrant B-catenin activation has been shown to suppress dendritic cell (DC) maturation, reducing antigen presentation and impairing T-cell priming (16, 17).

AML cells can also exploit metabolic pathways, such as increased glycolysis and lactate production, leading to an immunosuppressive metabolic microenvironment that inhibits T-cell activity. Increased glycolysis and subsequent lactate production lead to acidification of the tumor microenvironment, impairing T-cell function and promoting immune tolerance (18). Leukemic cells also exhibit increased consumption of essential metabolites such as glutamine and tryptophan, depleting the availability of key nutrients required for T-cell proliferation and effector function (19).

### 2.2 Tumor microenvironment-mediated resistance

The AML bone marrow microenvironment plays a critical role in immune evasion. From am immunological perspective, the AML microenvironment results in a complex intricate network of intrinsic and extrinsic factors recruited by neoplastic stem cells to induce a state of effective immunosuppression to drive disease progression. Remodeling of the microenvironment effectively inhibit successful anti-tumor immune responses, inducing an imbalance between T-effector cells and T-regulatory cells (Tregs), T-cell exhaustion through upregulation of immune checkpoint receptor and ligands, increased level of myeloid suppressor cells (MDSCs) and suppressive macrophage (M2), and an overall increased immunosuppressive state (2).

The presence of immunosuppressive cell populations, including myeloid-derived suppressor cells (MDSCs) and regulatory T cells (Tregs), inhibit effective T-cell responses. Additionally, AML cells can upregulate alternative immune checkpoints such as TIM-3 and LAG-3, bypassing PD-1/PD-L1 blockade. Pro-inflammatory cytokines such as TGF-β and IL-10 further contribute to immune suppression by inhibiting cytotoxic T-cell activity and promoting the expansion of suppressive immune cells. These factors collectively create an immune-suppressive niche that protects AML cells from immune-mediated destruction. Interferon gamma (IFN-γ) is a proinflammatory cytokine that can restore T-cell mediated anticancer immunity by increasing surface expression of HLA class II molecules thereby allowing recognition of AML cells by the immune system (20). It can have a dual role as IFN- is also capable of promoting PD-L1 and PDL 2 expression on AML cells (21). Recent studies show that IFN-γ has a complex role in modulating tissue microenvironment and in setting of monocytic AML, IFN-γ producing NK/T cells promotes an immunosuppressive environment that corelated with venetoclax resistance. In this context blocking IFN-γ signaling may be a therapeutic strategy (22).

The bone marrow niche further enhances immune resistance through complex interactions with AML cells. Mesenchymal stromal cells (MSCs) and fibroblasts in the bone marrow release cytokines like CXCL12 and VEGF, which suppress T-cell infiltration and promote the expansion of suppressive immune populations. Moreover, the hypoxic nature of the bone marrow environment favors immune evasion by stabilizing hypoxia-inducible factors (HIFs), which drive metabolic adaptations that inhibit T-cell function (23).

Zhang et al. studied single cell RNA transcriptomics data from bone marrow biopsy of AML patients and healthy controls. The investigators found that tissue resident memory T cells exhibited exhaustion phenotype with down-regulation of TCF7 and expression in CD8 positive cells. In solid tumors, similar downregulation of TCF7 is associated with reduced efficacy of CPI therapy. Simultaneously in the bone marrow biopsy specimens of AML patients, up-regulation of PD-1 in NK cell population was noted indicating exhaustion of NK cell subset. Despite having exhaustion signature, AML NK cells showed normal expression of cytotoxicity and cytokine secretion genes including *PRF1, IFNG, TNFG, CCL4* and *CCL5* (24). Gui et al. looked at single cell RNA sequencing data with spatial transcriptomics to study changes and used in bone marrow microenvironment and AML patients being treated with ICI and hypomethylating agent therapy. Patients that responded to ICI therapy showed enrichment of CD14 positive monocytes in the bone marrow which while the reticulocyte cells became more prevalent in nonresponders. The authors also described crosstalk (TWEAK signaling) between leukemia cells and adjacent dendritic cells naïve CD4 T cells and granulocyte monocytic progenitors post ICI therapy (25).

### 2.3 Genetic drivers and the bone marrow immune microenvironment

Acute myeloid leukemia (AML) arises within—then actively reshapes—the bone-marrow immune microenvironment. Recurrent driver lesions remodel the marrow niche by (i) altering cytokine and chemokine circuits, (ii) reprogramming antigen presentation and interferon responsiveness, (iii) skewing innate and adaptive cell composition (Tregs, MDSCs, macrophage polarization, NK impairment), and (iv) reshaping metabolism (lactate, adenosine, 2-HG) that enforces T-cell exhaustion. These programs act in leukemic blasts, LSCs, and stromal partners (mesenchymal stromal cells, osteolineage cells, endothelium, and sympathetic fibers), creating genotype-linked “immune ecotypes.”

Recurrent genomic drivers (e.g., FLT3-ITD, NPM1, IDH1/2, TP53, epigenetic modifiers) influence the composition and function of immune cells, antigen presentation, cytokine tone, and expression of immune checkpoints on blasts, leukemic stem cells (LSCs), and bystander immune cells. These alterations may potentially help determine response to ICIs and could be potentially integrated into combination therapy to improve responses (26, 27):

FLT3-ITD: Constitutive FLT3→STAT5 signaling drives glycolysis and PD-L1 transcription via histone lactylation, linking oncogenic signaling and tumor-intrinsic checkpoint upregulation. Histone lactylation at immune-regulatory loci directly upregulates PD-L1 expression on blasts and FLT3-ITD AML shows enrichment of exhausted T cell phenotype (PD-1, LAG-3, TIGIT). High PD-L1 expression in FLT3-ITD correlates with poor survival (6). FLT3-mut AMLs often display T-cell dysfunction and an immune-evasive milieu (28).
NPM1: NPM1-mutated AML can co-upregulate PD-L1—especially with concomitant FLT3-ITD—correlating with adverse outcomes; NPM1 creates neoepitopes that are exploitable for T-cell therapies but may coexist with exhausted T-cell states (29).
IDH1/2: The oncometabolite D-/R-2-hydroxyglutarate suppresses antitumor immunity by impairing T-cell migration and effector functions and by epigenetically reprogramming hematopoietic/immune cells; IDH inhibitors can reverse aspects of this immune paralysis, suggesting synergy with immunotherapy (30).
TP53 and chromatin modifiers: TP53-mut AML exhibits profound immune dysregulation, including defective antigen presentation and macrophage-mediated immune evasion (e.g., upregulated “don’t eat me” signals), contributing to poor outcomes and variable sensitivity to macrophage-engaging therapies.
DTA Epigenetic lesions (DNMT3A, TET2, ASXL1): Such epigenetic lesions rewire cytokine networks and antigen presentation programs that condition T-cell exhaustion and myeloid suppression (27, 31). Hypomethylating agents have been shown to be effective in re-expression of HLA and inducing “viral mimicry” to also upregulate PD-L1 (32).
RUNX1: RUNX1 mutations impair dendritic maturation and costimulatory molecule expression. AML with RUNX1 mutations shows enrichment of plasmacytoid dendritic cell signatures but poor antigen presentation, accompanied by T cells expressing TIM-3 and LAG-3 (17, 33).
KMT2A (MLL) rearrangements: KMT2A-rearranged AML exhibits high HOXA/MEIS1 activity with monocytic polarization, strong TIM-3 expression on blasts, and exhausted T-cell infiltrates, suggesting a mixed “immune-hot but exhausted” state (34).

AML driver mutations fundamentally reshape the bone marrow immune landscape and regulate checkpoint molecule expression. Recognizing these genotype-immune interactions is essential for rational trial design and the development of precision immunotherapy in AML.

### 2.4 Host-related factors

Beyond tumor-intrinsic and microenvironmental factors, patient-specific factors influence checkpoint inhibitor responses. Pre-existing immune exhaustion due to prior treatments, age-related immune dysfunction, and clonal hematopoiesis can impair the efficacy of immunotherapies (35). Additionally, genetic predispositions and polymorphisms affecting immune regulatory pathways may influence treatment outcomes.

The composition and functionality of a patient’s immune repertoire also play a role in therapy response. Patients with a pre-existing exhausted T-cell phenotype, characterized by high expression of inhibitory receptors and reduced effector function, are less likely to respond to checkpoint blockade. Moreover, individual variations in gut microbiota have been implicated in modulating systemic immune responses, potentially influencing the effectiveness of immunotherapies (36).

## 3 Clinical trial data on checkpoint inhibitor therapy in AML

The presence of immune checkpoint interactions in AML, both at diagnosis and in the relapsed setting, has led to significant interest in using this therapeutic approach to reactivate immune sensitivity through blockade of checkpoint co-inhibitory ligands. Despite the encouraging activity of checkpoint inhibitor therapy in solid tumors, the activity in AML has led to limited success, attributed to be secondary to the low mutational burden and DNA mismatch repair proficiency in AML compared to solid tumors (37, 38). In AML, bone marrow infiltrating T-cell populations are preserved and even increased compared to healthy individuals, often with increased frequency of immune inhibitory and activating co-receptor expression, including PD-1, OX40, TIM3, and LAG3 and this has led to interest in the potential of ICIICIs in AML (12, 39). The available clinical data for ICIICIs in AML evaluated in clinical trials will be discussed in this review and are further summarized in **Table 1**.

**Table 1:** Summary of selected trials evaluating immune checkpoint inhibitors in treatment of AML.

Target	Agent	Regimen	Study population	Phase	NCT identifier	Status
PD-1	Nivolumab	Nivolumab	High risk AML	II	NCT02532231	Completed
		Nivolumab + AZA	R/R AML	Ib/II	NCT02397720	Completed
		Nivolumab + IC	ND AML	Ib/II	NCT02464657	Recruiting
		Nivolumab + Ipilimumab	R/R AML MDS following allo-HSCT	Ib	NCT03600155	Completed
		Nivolumab	Post allo-HSCT with PT-Cy	I	NCT04361058	Withdrawn
		Nivolumab and oral cyclophosphamide	R/R AML	II	NCT03417154	Completed
	Pembrolizumab	Pembrolizumab + AZA	R/R AML Newly diagnosed	II	NCT02845297	Completed
		IC ± Pembrolizumab (BLAST MRD AML-1)	ND AML	II	NCT04214249	Active, not recruiting
		AZA + VEN + Pembrolizumab (BLAST MRD AML-2)	ND AML	II	NCT04284787	Active, not recruiting
		DEC + Pembrolizumab ± VEN	R/R AML	Ib/II	NCT03969446	Recruiting
		DEC + Pembrolizumab	ND AML R/R AML post allo-HSCT	II	NCT03092674	Terminated
		Pembrolizumab	Relapse post allo-HSCT	I/II	NCT03286114	Terminated
		Pembrolizumab	Relapse post allo-HSCT	I/II	NCT02981914	Completed
		Cytarabine followed by Pembrolizumab	R/R AML	II	NCT02768792	Completed
CTLA-4	Ipilimumab	Ipilimumab + decitabine	R/R AML	I	NCT02890329	Active, not recruiting
		Ipilimumab	Post allo-HCT	I	NCT02846376	Terminated
CD47-SIRPa	Magrolimab	AZA + VEN + Magrolimab	ND AML R/R AML	Ib/II	NCT04435691	Terminated
		AZA + Magrolimab *vs*. Placebo (ENHANCE)	ND HR-MDS	III	NCT04313881	Terminated
		AZA + Magrolimab *vs*. AZA/VEN or IC (ENHANCE-2)	ND TP53-mutated AML	III	NCT04778397	Terminated
		AZA + VEN + Magrolimab *vs*. Placebo (ENHANCE-3)	ND AML	III	NCT05079230	Terminated
TIM-3	Sabatolimab	AZA + VEN + Sabatolimab (STIMULUS-AML1)	ND AML	II	NCT04150029	Completed
		Sabatolimab ± AZA	MRD+ AML post allo-HSCT	Ib/II	NCT04623216	Completed

AZA, azacitidine; DEC, decitabine; VEN, venetoclax; IC, intensive chemotherapy; ND, newly diagnosed; R/R, relapsed/refractory.

### 3.1 CTLA-4

CTLA-4 (CD152) is an immune checkpoint receptor that interacts with CD80 and CD86 to suppress immune responses, regulate tolerance, and influence autoimmunity (40). In AML, abnormal CTLA-4 expression has been linked to worse disease outcomes (41). Preclinical studies show that blocking CTLA-4 enhances T-cell activity and reduces regulatory T cells (Tregs), potentially improving anti-tumor responses and this subsequently led to its clinical exploration (42). Early studies in AML cell lines and preliminary clinical trials suggested improvement in T-cell frequency, cytotoxicity, and IFN-γ secretion (43).

However, clinical responses in AML patients have been inconsistent, with most of the clinical investigation for ipilimumab has been in the relapse post-HCT setting. Trials testing ipilimumab as a single agent in relapsed hematological malignancies post-HCT reported complete and partial response rates of 23% and 9%, respectively (44). Another study by Bashey et al. reported essentially no response in a study of 29 patients relapsed after HCT (45). Careful assessment of risk and benefit needs to be done when ipilimumab is used in the posttransplant setting due to risk of immune related adverse events and flareup of GVHD which can be seen with these drugs (44). A phase I trial is currently evaluating ipilimumab in combination with Treg-depleted donor lymphocyte infusion (DLI) in AML and MDS patients who relapsed post-HCT (NCT03912064). A phase I trial is investigating the combination of ipilimumab with decitabine (DAC) in relapsed or refractory AML/MDS, though recruitment is currently inactive (NCT2890329).

### 3.2 PD-1/PDL1 axis

PD1 (CD279) is a transmembrane protein expressed on activated immune cells that interacts with two ligands, PD-L1 (CD274) and PD-L2 (CD273), that are often upregulated in AML and correlate with immune suppression, therapy resistance, and poor prognosis (46, 47). PD1 overexpression in CD8+ T-cells has been associated with T-cell dysfunction and disease progression (48). Additionally, the co-expression of PD1 and T-cell immunoglobulin and mucin domain 3 (TIM-3) on T-cells has been linked to T-cell exhaustion and leukemia relapse post-HCT, with higher frequencies observed in patients experiencing multiple relapses (49, 50). Increased PD-L1/PD-L2 expression at diagnosis, relapse, and during treatment has been linked to reduced therapeutic response and worse outcomes (51, 52). The PD1/PD-L1 interaction contributes to immune evasion by promoting T-cell exhaustion and apoptosis while enhancing Treg differentiation and tumor cell resistance to CD8+ T-cell cytolysis (53).

Three PD1-blocking antibodies (nivolumab, pembrolizumab, and cemiplimab) and three PD-L1 inhibitors (atezolizumab, avelumab, and durvalumab) have been approved for treatment in various solid tumors, but none have been FDA-approved for AML to date. However, several clinical trials are investigating their efficacy. Nivolumab has been tested as a maintenance therapy in high-risk AML and for eliminating MRD, but results have been mixed (54). A randomized phase 2 CTEP trial (NCT02275533) looking at nivolumab versus placebo maintenance in high-risk AML patients who are not candidates for HCT fail to show improvement in overall survival or leukemia free survival. More adverse events were seen in the study arm, but these were expected and manageable with supportive care (55). An ongoing phase II trial is evaluating nivolumab in relapsed AML and MRD-positive AML post-HCT (NCT03146468), while a phase I/Ib trial with similar design was terminated due to early toxicity. Pembrolizumab has been examined as a maintenance therapy in elderly AML patients and as a salvage post-transplant therapy.

Clinical trials are also evaluating combinations of ICIs with epigenetic therapies (133). Hypomethylating agents (HMAs) have been shown to upregulate immune checkpoint receptors, including CTLA-4 and PD-1, in AML models. Clinical studies indicate that azacytidine (AZA) can induce PD-1 promoter demethylation, leading to increased PD-1 expression and poorer outcomes in the absence of immune checkpoint blockade (121). Initial data suggests that patients previously treated with hypomethylating agents (HMAs) have lower overall response rates (22%) compared to HMA-naïve patients (58%), indicating that combining HMAs with ICIs may be more effective when introduced earlier in treatment. In the NCT02397720 trial by Daver et al., which studied AZA plus nivolumab or AZA plus nivolumab with ipilimumab in relapsed/refractory AML or elderly newly diagnosed AML patients, reported overall response rates (ORR) of 44% and complete/partial remission rates of 36% in the triple therapy group. In NCT02845927, the combination of AZA and pembrolizumab in relapsed/refractory AML and newly diagnosed patients over 65 showed modest clinical activity (56). Similarly, a trial assessing pembrolizumab with decitabine (NCT02530463) indicated potential benefits in relapsed/refractory AML. In another Phase 1b/II trial (NCT03969446), the impact of pembrolizumab in R/R AML is being evaluated in combination with 10-day course of decitabine and in combination with 10-day course of decitabine plus venetoclax. This study is of unique interest demonstrating potential response after patients with R/R AML have failed prior hypomethylating agent therapy – in reported data of 6 patients, a 67% overall response rate was observed (57).

In a meta-analysis of 13 studies of ICI therapy in AML, overall response rate (ORR) has been 42% with complete remission (CR)/response with incomplete hematologic count recovery (CRi) rates of 33%. Efficacy results did seem to demonstrate improved response in the first-line setting, with average OS in front-line being 12 months and OS in the relapsed/refractory (R/R) setting being 7.3 months. The incidence of immune-related adverse events ≥ grade 3 (irAEs) was low and estimated to be about 12% across studies (58).

Other studies have examined nivolumab combined with chemotherapy. A phase II trial of nivolumab, cytarabine, and idarubicin in newly diagnosed AML/MDS (NCT024645657) found that responders minimal residual disease (MRD) clearance, while non-responders frequently had TP53 mutations and higher bone marrow CD4+ cells co-expressing PD1 and TIM-3 (59). Another phase II trial (NCT02768792) is assessing pembrolizumab with high dose cytarabine (HiDAC) in relapsed/refractory AML, showing that pretreatment CD8+ TCR diversity correlates with response. Preliminary results indicate that increased expression of chemokine receptors in CD8+ cells and genes involved in p53, IFN-γ, and IL-6 pathways may predict better outcomes (60).

To date, there has not been any trial investigating the efficacy of cemipilimab in AML. The combination of AZA and durvalumab in patients with AML and MDS unfit for intensive chemotherapy has shown promising results, with an ORR of 61.9% and median overall survival of 11.6 months in MDS and ORR 31% with median OS 13 months in AML (61). Several phase I and II trials are investigating PD-L1 inhibitors, such as avelumab and atezolizumab, in combination with HMAs (AZA, DAC, or guadecitabine), but no results are currently available (NCT02953561, NCT03395973, NCT02892318, NCT029355361, NCT3154827).

### 3.3 TIM-3/Galectin-9 pathway

TIM-3 is an inhibitory receptor present on CD4+ Th1 cells, CD8+ cytotoxic T lymphocytes (CTLs), and various innate immune cells, including dendritic cells, monocytes, macrophages, mast cells, and NK cells. It is also expressed on certain malignant cells (62).

Among the four known TIM-3 ligands, galectin-9 (gal-9) is the most studied, primarily due to its role in promoting Th1 cell apoptosis and facilitating tumor immune evasion (63–65). Increased TIM-3 expression has been linked to T-cell dysfunction in both human and murine cancer models. Frequently co-expressed with PD-1, inhibiting TIM-3 alone or alongside other immune checkpoints has demonstrated the potential to restore T-cell function. In AML, TIM-3 is highly expressed on immune cells, especially T-cells and NK cells, where it contributes to immune exhaustion (66). Additionally, it serves as a distinct marker for leukemic stem cells (LSCs), which do not exhibit TIM-3 in normal hematopoietic stem cells (66, 67).

Elevated TIM-3 expression correlates with poor prognosis in both solid and hematologic malignancies (52). Furthermore, TIM-3 overexpression has been associated with negative outcomes in AML, with TIM-3 overexpression observed in CD4+ T-cells from FLT3-mutated AML and in CD8+ cells of patients with high-risk AML (68). TIM-3 upregulation has also been associated with negative prognostic values in AML with normal cytogenetics (69). Kong et al. has additionally demonstrated shorter leukemia-free survival after allo-HCT consolidation in patients with high number of TIM-3/PD-1 co-expressing T-cells, implicating the role of TIM-3 as a potential target to prevent post-HCT relapse (49).

Given its significant role in AML progression, TIM-3 is being explored as a therapeutic target for monoclonal antibody (MoAb) treatments. Blocking TIM-3/gal-9 interactions *in vitro* has demonstrated reduced AML cell proliferation (70). Murine models suggest that anti-TIM-3 MoAbs can eradicate LSCs without affecting normal hematopoiesis.

Several MoAbs targeting TIM-3 are under investigation in clinical trials for solid tumors, including sabatolimab (MBG453), TSR-022, BMS-986258, LY3321367, SYM023, BGB-A425, and SHR 1702. Among these, sabatolimab has shown promising early efficacy in AML and MDS. Ongoing clinical trials are evaluating sabatolimab alone and in combination with HMAs, PD-1 inhibitors, the MDM2 inhibitor HDM201, and venetoclax. Preliminary results from the NCT03066648 trial, which tested sabatolimab with HMAs, reported an overall response rate (ORR) of 58% in MDS and 38% in newly diagnosed AML patients for the DAC arm, while the AZA arm demonstrated ORRs of 70% and 27%, respectively. The most common severe adverse events (AEs) included thrombocytopenia, anemia, and neutropenia, with a few immune-related events observed in the DAC-treated cohort (71, 72).

### 3.4 LAG-3/MHC axis

LAG-3 (CD223) is a receptor structurally similar to CD4 that binds MHC class II molecules with higher affinity than CD4, thereby suppressing T-cell activation. LAG-3’s primary ligand is MHC-II, which competes with CD4 binding to downregulate T-cell responses. Beyond MHC-II, LAG-3 can also bind Galectin-3 (Gal-3), a lectin expressed in tumors and activated T-cells. Gal-3/LAG-3 interactions suppress CD8+ T-cell cytotoxic activity and inhibit plasmacytoid dendritic cell expansion, reducing anti-tumor immune responses. Another key ligand is fibrinogen-like protein 1 (FGL1), a member of the fibrinogen family induced by IL-6, which is overexpressed in various cancers and has been associated with resistance to PD-1 blockade therapies (42, 73, 74).

LAG-3 is present on activated CD4+ and CD8+ T-cells, regulatory T-cells (Tregs), NK cells, B-cells, and dendritic cells. It is particularly expressed in Th1 and Th0 subsets but absent in Th2 cells. In the tumor microenvironment, LAG-3 is frequently co-expressed with PD-1 on exhausted CD8+ T-cells (52). While its role in immune suppression is well-documented in solid tumors, its function in AML remains unclear.

In solid tumors, LAG-3 and PD-1 co-expression has been linked to reduced responsiveness to PD-1 inhibitors, making it a potential biomarker for predicting immunotherapy efficacy (75). However, its role in AML requires further investigation, particularly given the involvement of MHC-II in both immune suppression and antigen presentation in AML blasts (76–81). Antibodies targeting LAG-3 are currently under evaluation for various malignancies, including solid tumors, lymphomas, and multiple myeloma, often in combination with PD-1 inhibitors. In AML, only one clinical trial, the AARON study (NCT04913922), has been initiated to assess the safety and efficacy of a combination therapy involving AZA, nivolumab (anti-PD-1), and relatlimab (anti-LAG-3) in relapsed/refractory AML and newly diagnosed AML patients over 65 years of age. Recruitment for this trial began in November 2022, but results are not yet available.

### 3.5 The TIGIT-CD155/CD112 pathway

TIGIT is a protein encoded by a gene located on chromosome 3q13.31. It comprises an extracellular IgV domain, a transmembrane (TM) region, and a cytoplasmic tail containing an immunoreceptor tyrosine-based inhibitory motif (ITIM) and a tail-tyrosine (ITT)-like phosphorylation motif. This protein is specifically expressed on T-cells and natural killer (NK) cells (82). TIGIT interacts with CD155 and CD112, showing varying binding affinities, and exerts an immunosuppressive role by transmitting inhibitory signals (83). It competes with CD226 and CD96, which provide costimulatory signals. Initially described by Yu et al. in 2009, TIGIT was recognized as an immune checkpoint that inhibits T-cell activation by facilitating the maturation of immunoregulatory dendritic cells (DCs). Beyond its indirect function through DCs, TIGIT engagement directly suppresses T-cell and NK cell activity (82, 84).

CD155 expression is typically low in normal tissues but is significantly upregulated in various malignancies, including melanoma, pancreatic cancer, lung adenocarcinoma, colon cancer, and glioblastoma. Its presence has been linked to tumor progression, invasion, and unfavorable clinical outcomes through interaction with TIGIT. In AML, however, the role of TIGIT remains unclear (85). Research by Kong et al. indicated that TIGIT expression on peripheral T-cells in AML patients correlated with CD8+ T-cell exhaustion and poorer prognosis (86). Liu et al. identified an association between TIGIT expression and impaired NK cell function in AML (87). Furthermore, TIGIT frequently coexists with other inhibitory receptors such as PD-1, TIM-3, and LAG-3, leading to diminished CD8+ cytotoxic T-cell function. Studies have also connected TIGIT expression on peripheral CD4+ T-cells following hematopoietic cell transplantation (HCT) with disease relapse. Additionally, the overexpression of TIGIT ligands on leukemic cells might facilitate immune evasion. Laboratory research on AML cell lines and primary AML samples suggests that targeting the TIGIT-CD155/CD112 axis could enhance anti-leukemia immune responses. Therefore, blocking this pathway using monoclonal antibodies presents a promising therapeutic avenue. While numerous clinical trials are evaluating anti-TIGIT antibodies for solid tumors, there are currently no active trials investigating this approach in AML.

### 3.6 The CD47/SIRPα pathway

CD47, also known as integrin-associated protein, is a transmembrane protein encoded by a gene located on chromosome 3q13.12. It functions as an anti-phagocytic “do not eat me” signal, allowing CD47-expressing cells to evade macrophage-mediated clearance. This inhibition of phagocytosis is mediated through CD47’s interaction with signal regulatory protein alpha (SIRPα) on macrophages and DCs. This binding triggers a signaling cascade that activates tyrosine phosphatases, preventing the accumulation of myosin at the phagocytic synapse and thereby inhibiting phagocytosis. Initially identified in red blood cells, CD47 is now known to be widely expressed across many normal cell types. In oncology, CD47 was first discovered in human ovarian cancer in the late 1980s and has since been identified in numerous solid tumors and hematologic malignancies. Currently, CD47 is recognized as a universal immune evasion mechanism utilized by cancer (88, 89).

In AML, high CD47 expression on the cell surface and elevated CD47 mRNA levels have been associated with poor prognosis (88). Furthermore, CD47 levels are notably elevated in leukemic stem cells (LSCs) characterized by the CD34+CD38-CD90-lin- phenotype, distinguishing them from normal counterparts and making CD47 a potential target for anti-leukemic therapies. Preclinical studies using anti-CD47 antibodies have demonstrated selective depletion of LSCs, and murine models have confirmed that leukemia fails to engraft in secondary recipients following treatment, indicating LSC depletion. However, as CD47 is also broadly expressed on normal cells, off-target toxicity remains a concern. Comparisons between normal and cancerous cells reveal that while both evade phagocytosis via CD47, only malignant cells also express pro-phagocytic signals. Blocking CD47 activates “eat me” signals specifically in cancer cells. Chao et al. identified calreticulin as a key pro-apoptotic signal that enhances phagocytosis in cancer cells (90). Additionally, inhibiting the CD47/SIRPα pathway promotes macrophage polarization toward an M1 phenotype, enhances macrophage recruitment, and facilitates antigen presentation to CD8+ T-cells, thus inducing an adaptive anti-tumor immune response (91). Blocking this axis also enhances NK-mediated antibody-dependent cytotoxicity (ADCC), complement-mediated cytotoxicity, and cancer cell apoptosis while reducing tumor proliferation and migration (92).

Given these findings, anti-CD47 monoclonal antibodies have been developed for clinical applications. CC-90002 was the first-generation humanized anti-CD47 antibody to enter clinical trials. Despite promising preclinical results, a Phase I study in patients with relapsed/refractory AML and myelodysplastic syndromes (MDS) was discontinued due to insufficient efficacy as monotherapy, with none of the 28 enrolled patients deriving significant benefit (93). The lack of efficacy was attributed to the switch from IgG1 to IgG4, which significantly reduced its cytotoxic potential. Research into CD47-targeting therapies for hematological malignancies was subsequently halted.

In 2020, however, the FDA granted breakthrough therapy designation to magrolimab, another anti-CD47 antibody, based on promising efficacy data in MDS. In high-risk MDS, a Phase 1b study combining magrolimab with azacitidine (AZA) demonstrated a favorable safety profile, with the most common treatment-emergent adverse events (TEAEs) including constipation (68%), thrombocytopenia (55%), anemia (52%), neutropenia (47%), nausea (46%), and diarrhea (44%). Response rates were encouraging, with a complete response (CR) rate of 33% and an overall response rate (ORR) of 76%. The overall survival (OS) probability was 75% at 12 months and 52% at 24 months. Similar outcomes were observed in patients with TP53 mutations (94). In TP53-mutated AML, a Phase 1b trial combining magrolimab with AZA reported an ORR of 48%, including a CR rate of 33.3% (216). A Phase 3 trial comparing magrolimab plus AZA against standard care in TP53-mutated AML is underway (ENHANCE2, NCT04778397), along with another Phase 3 randomized trial evaluating AZA plus magrolimab or placebo in MDS (ENHANCE trial—NCT04313881). Another clinical trial evaluating magrolimab added to combination of azacitidine *vs*. azacitidine + venetoclax *vs*. oral azacitidine is ongoing (NCT04778410).

It is important to note that in 2022, the FDA temporarily halted trials assessing magrolimab plus AZA due to inconsistencies in reporting severe adverse events. The trials resumed following a reassessment of safety data, but the interruption negatively impacted several studies. Currently, additional combinations involving magrolimab are being explored in MDS and AML, including magrolimab plus anti-PD-L1 atezolizumab (NCT03922477), magrolimab plus AZA plus venetoclax (NCT0443691), and magrolimab with other agents such as daratumumab, pomalidomide, dexamethasone, and bortezomib (NCT04892446).

Newer CD47-targeting agents are also in development. Evorpacept (ALX148) is a high-affinity CD47-blocking protein with a modified Fc domain that avoids red cell agglutination. Ongoing trials are evaluating ALX148 with AZA in high-risk MDS (ASPEN02 trial—NCT04417517) and in combination with AZA plus venetoclax for AML (ASPEN05 trial—NCT04755244). Preliminary Phase 1 results suggest that the ALX148-AZA combination is well tolerated, with mild TEAEs and promising anti-leukemic activity (95). Additionally, lemzoparlimab, a second-generation IgG4 anti-CD47 antibody with red-cell-sparing properties, is being evaluated in relapsed/refractory AML and MDS (NCT04202003) (96).

Clinical data on emerging anti-CD47 antibodies is incomplete at present, and the development of new molecules targeting the CD47/SIRPa axis including bispecific antibodies and SIRPa/Fc fusion protein antibodies are also under ongoing investigation to define the therapeutic potential of these emerging CD47/SIRPα inhibitors.

### 3.7 NK cell checkpoint blockade

Apart from the well-described immune checkpoints in T-cell population, NK cell receptors are now increasingly being described as immune checkpoints. These NK cell receptors include killer immunoglobulin like inhibitor receptors (KIRs), C type lectin like inhibitory receptors such as natural killer group 2 NKG2A/CD94 complex and leukocyte immunoglobulin type receptor (LILRs). NK cells express cell surface receptors that recognize MHC class I alleles. KID, CD94/NKG2A and LILRB1 R remain elevated receptors involved in self HLA class I LDL recognition. Activation of this inhibitory receptor results and inhibitory signals that impedes NK cell mediated cell lysis. In animal models’ presence of anti-NKG2A antibodies enhances tumor elimination pattern use in combination with anti-PD-1 or anti EGFR antibodies. This effect may be seen as binding of NKG2A/CD94 to MHC class I molecules results and inhibition of effective functions of NKG2A expressing NK and T cells (97).

The role of killer immunoglobulin like inhibitory receptors (KIR) in haploidentical hematopoietic stem cell transplantation was pioneered by Ruggeri et al, demonstrating that donor-recipient KIR-HLA mismatches can prevent leukemia relapse and protect patients against GVHD (98). The major determinant of NK cell allo reactivity is reduced expression of KIRs on donor NK cells that do not recognize HLA C on recipient tissues. Donor NK cells lacking KIR-HLA-C mediated inhibition, can differentiate and expand in the recipient and mediated potent anti-AML activity preventing post-transplant relapse. These activated donor NK cells and eliminate recipient T cells and dendritic cells resulting in superior engraftment and reduction in GVHD. Humanized monoclonal antibody ipilimumab is being used in clinical trials in patients with multiple myeloma and AML. Early results show safety and feasibility and treated patients (44, 45). *In vivo* administration of this antibody results in complete occupancy of KIR receptors leading to reduction of KIR2D expression on circulating NK cells, a process mediated by trogocytosis of KIR2D by monocyte macrophage cells in peripheral blood or liver spleen. This eventually resulted in cytotoxicity of tumor cells expressing HLA-C by circulating NK cells. Thus, inhibition of checkpoint blockage of NK cells mediated by NKG2A/CD94 and KIR receptors using monoclonal antibody could result in therapeutic responses in patients with hematologic malignancies.

## 4 Strategies to overcome resistance to ICI therapy in AML

**Figure 2:**
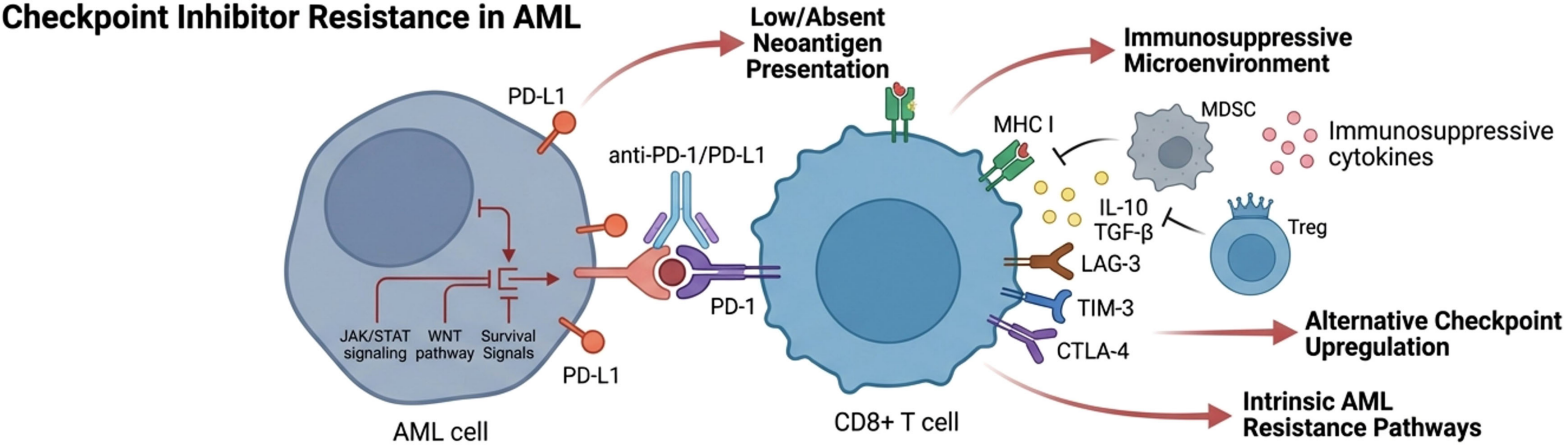
Schema of checkpoint inhibitor in AML.

**Figure 3:**
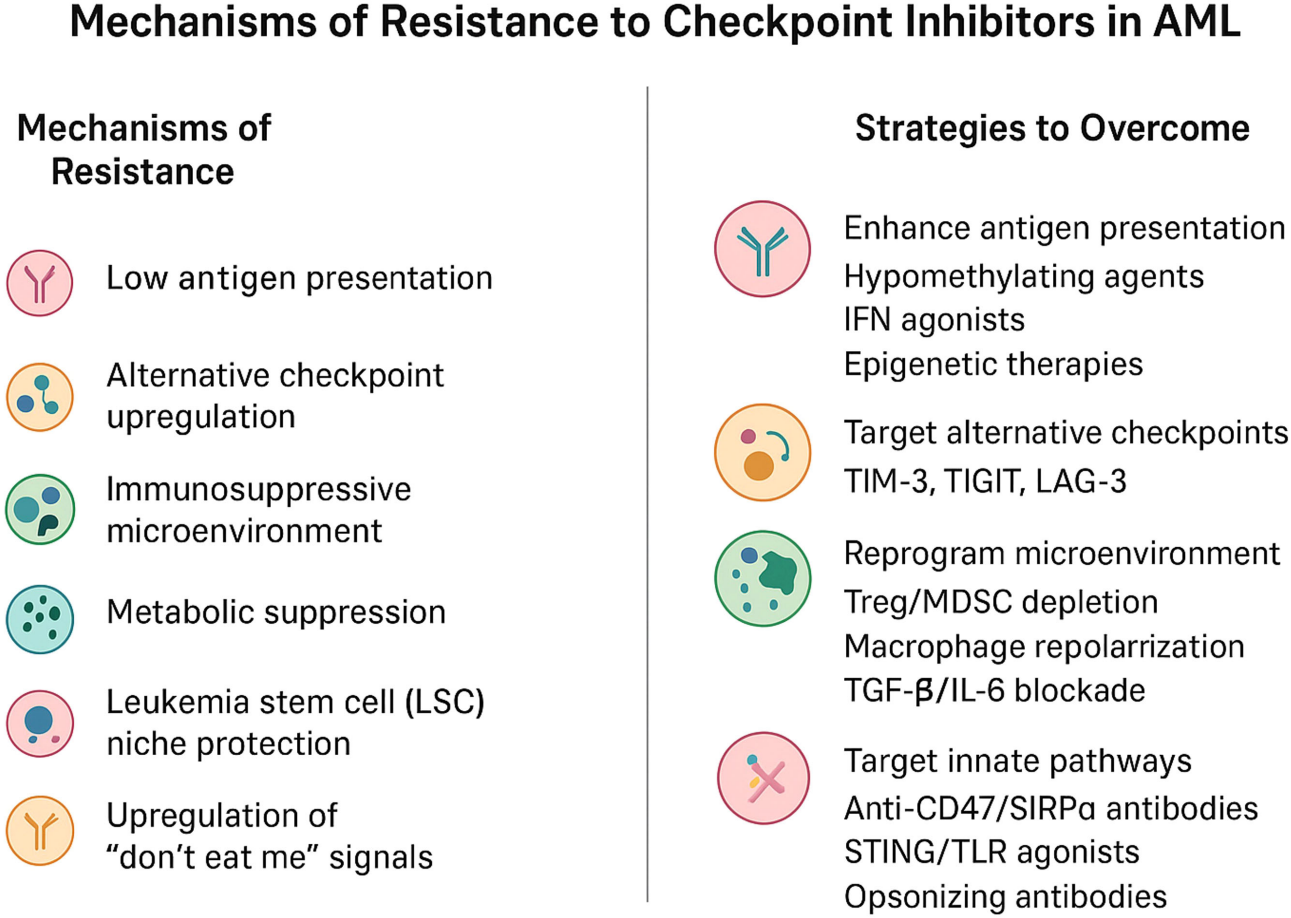
Summary of mechanism or resistance to checkpoint inhibitors and strategies to overcome resistance.

Immune checkpoint inhibitor (ICI) therapy has demonstrated limited clinical efficacy in acute myeloid leukemia (AML), largely due to intrinsic and microenvironment-driven mechanisms of immune resistance (**Figure 2**). Emerging strategies to overcome this resistance increasingly emphasize rational combination approaches that target multiple immunosuppressive pathways simultaneously. Combination therapies pairing ICIs with hypomethylating agents, targeted therapies, or cellular immunotherapies have shown potential to enhance antigen presentation and sensitize leukemic cells to immune-mediated killing. Reversal of T-cell exhaustion through modulation of inhibitory receptor signaling, epigenetic reprogramming, and cytokine support represents a complementary strategy to restore effector function. In parallel, therapeutic modulation of the immune microenvironment—particularly targeting myeloid-derived suppressor cells, regulatory T cells, and immunosuppressive cytokine networks—aims to dismantle dominant barriers to antitumor immunity in the bone marrow niche. Finally, metabolic interventions that restore nutrient availability and disrupt immunosuppressive metabolic programs, including amino acid depletion and aberrant lipid metabolism, are emerging as promising adjuncts to checkpoint blockade. Together, these multifaceted strategies highlight a shift toward integrated immunotherapeutic regimens designed to recondition immune competence and improve ICI responsiveness in AML (**Figure 3**).

### 4.1 Combination therapies

Combining checkpoint inhibitors with other therapeutic agents has shown promise in overcoming resistance (99). Hypomethylating agents (HMAs) such as azacitidine and decitabine can enhance the expression of immune-related genes, improving antigen presentation and T-cell activation. The combination of checkpoint blockade with immune-stimulatory agents such as IL-15 or CD40 agonists has also been explored to enhance antitumor immunity. Additionally, targeted therapies such as FLT3 inhibitors may synergize with immune checkpoint inhibitors by modulating immune cell infiltration and activation.

Combination of ICIs with traditional chemotherapy is one of the most widely explored strategies. Chemotherapy, although still the backbone of AML treatment, is known to induce tumor antigen release and promote T-cell priming. Thus, combining chemotherapy with checkpoint blockade could enhance the immune response. In early-phase clinical trials, combining nivolumab or pembrolizumab (anti-PD-1) with chemotherapy agents like cytarabine has been shown to improve responses in some AML patients, particularly those with relapsed or refractory disease (100). ICI therapy can also be combined with bispecific T-cell engagers (BiTE) which are antibody based molecules composed of 2 single chain fragment variable domains 1 specific for tumor antigen and the other one for CD3 on a single polypeptide chain (101). AMG 330 was developed in patients with AML and simultaneously engages CD33 positive AML blasts and CD3 positive T cells. Clinical efficacy was low given up regulation of PD-1, TIM3 and LAG-3 on CD3 positive T cells resulting in promotion of an exhausted phenotype of T-cells. Blockade of PD-1/PD-L1 pathway is associated with significant increase in AMG 330 mediated T-cell proliferation interferon gamma secretion and tumor lysis (102, 103). Similarly, combining checkpoint inhibitors with targeted therapies such as FLT3 inhibitors (e.g., midostaurin, gilteritinib, quizartinib) and IDH inhibitors (e.g., ivosidenib, olutasidenib) offers the potential for synergistic effects. FLT3 mutations are common in AML and have been shown to contribute to immune evasion, with FLT3 inhibitors enhancing the immune response. By combining FLT3 inhibitors with ICIs, researchers hope to overcome the immune suppressive effects of these mutations and re-activate the immune system.

The STING (stimulator of interferon genes) is another pivotal pathway instrumental in activating innate immunity and has emerged as a novel target in cancer immunotherapy. Some STING agonists have been shown to have a key role in activating NK cells and increase the recruitment and activity of T cells within the tumor microenvironment, essentially converting an immunologically “cold” tumor to an immunologically “hot” tumor, rich in activated immune cells (104). There has been interest in overcoming ICI resistance using combination therapy with STING agonists and this been previously demonstrated in a lung metastasis model as well as in high-grade serous ovarian cancer (105, 106). There are several ongoing clinical trials evaluating the combination of STING agonists with ICI therapy in various different tumor types in pre-clinical models (107–109). A fist-in-clinic Phase I study (NCT05424380) is evaluating GSK3745417 in R/R AML and high—risk MDS, offering potential interest for future studies in combination with ICIs.

### 4.2 Reversing T cell exhaustion

Anti-PD-1 antibodies block PD-1 signaling, thereby restoring T cell receptor-mediated activation and preventing apoptosis. Similarly, anti-CTLA-4 antibodies reduce regulatory T cell (Treg) populations within the tumor microenvironment (TME) and modulate T cell receptor diversity. These mechanisms can help rejuvenate exhausted T cells. However, alternative immune checkpoints continue to contribute to T cell exhaustion, meaning that ICIs alone may not completely reverse this state (110).

T cell immunoglobulin and mucin-domain containing-3 (TIM-3) is an inhibitory receptor expressed on IFN-γ-producing T cells, certain Treg subsets, and other innate immune cells (111). TIM-3 activation suppresses Th1 cells, reduces the production of pro-inflammatory cytokines such as TNF and IFN-γ, and impairs cytotoxic T cell function. High TIM-3 expression is a hallmark of T cell exhaustion (111). Current research is evaluating combinations of ICIs with anti-TIM-3 antibodies.

While the most progression of clinical investigation has been investigation of TIM-3 inhibition, there has additionally been interest in LAG-3 and TIGIT. Other inhibitory receptors, such as VISTA, B7-H3, and BTLA, are also being examined in clinical trials (112, 113). In addition to blocking co-inhibitory pathways, research is exploring the activation of co-stimulatory receptors, including CD137, glucocorticoid-induced TNFR-related protein (GITR), OX40, and CD27, to enhance T cell proliferation and activation (113). Several agonist-based therapies targeting these pathways are currently under investigation, either alone or in combination with ICIs.

Beyond ICIs, tumor-infiltrating lymphocyte (TIL) therapy is showing potential in reversing T cell exhaustion. TIL therapy involves isolating and expanding autologous immune cells ex vivo before reinfusing them into patients, enhancing their ability to target tumor-associated antigens (114).

### 4.3 Modulating the immune microenvironment

Altering the AML immune microenvironment is another approach to enhancing checkpoint inhibitor efficacy. Strategies such as depleting MDSCs and Tregs using small molecules or monoclonal antibodies can enhance the immune response. Dendritic cell-based vaccines and immune-modulatory drugs like lenalidomide can improve antigen presentation and stimulate T-cell responses. Targeting alternative immune checkpoints such as TIM-3 and CD47 also holds promise in overcoming immune evasion mechanisms. AML’s immune suppressive microenvironment is a major contributor to resistance to checkpoint blockade. This microenvironment is rich in regulatory T-cells (Tregs), myeloid-derived suppressor cells (MDSCs), and tumor-associated macrophages (TAMs), all of which inhibit anti-tumor immune responses. Strategies aimed at modulating the tumor microenvironment could improve the effectiveness of ICIs. Targeting and depleting immune-suppressive cells such as Tregs and MDSCs could restore immune function in AML patients. Several strategies are being explored, including using monoclonal antibodies to target specific markers on these cells. For example, anti-CD25 antibodies can be used to selectively deplete Tregs, while targeting the immunosuppressive molecules expressed by MDSCs, such as arginase-1, could enhance T-cell function (115).

TAMs in the tumor microenvironment often express immunosuppressive molecules such as PD-L1, TGF-β, and IL-10, which inhibit T-cell activation and promote tumor survival. Targeting TAMs with small molecules or antibodies that block the CSF-1R (colony-stimulating factor 1 receptor) or other signaling pathways may reduce their immunosuppressive effects. Additionally, targeting macrophages to shift their polarization from an M2 (pro-tumoral) to an M1 (anti-tumoral) phenotype is an area of ongoing research (116).

Among the key players contributing to ICI resistance are myeloid-derived suppressor cells (MDSCs) and regulatory T cells (Tregs), which suppress effector T-cell activity. Additionally, dysregulated cytokine signaling in the AML TME further exacerbates immune dysfunction. MDSCs are a heterogeneous population of immature myeloid cells that accumulate in the TME and actively suppress antitumor immunity. These cells inhibit cytotoxic T-cell responses through several mechanisms, including metabolic interference, direct suppression via immune checkpoint ligand expression, and cytokine secretion. One of the primary mechanisms of immune suppression by MDSCs is the depletion of L-arginine through the activity of arginase-1, an enzyme that depletes a key amino acid necessary for T-cell function (117). Additionally, MDSCs generate reactive oxygen species (ROS) and nitric oxide, which further impair T-cell receptor (TCR) signaling and promote T-cell exhaustion.

In AML, MDSCs contribute significantly to ICIICI resistance by maintaining a highly immunosuppressive TME. Their ability to express programmed death-ligand 1 (PD-L1) provides an additional mechanism of immune evasion by directly inhibiting effector T-cell function (118). Furthermore, MDSCs support the expansion of Tregs by producing immunosuppressive cytokines such as transforming growth factor-beta (TGF-β) and interleukin-10 (IL-10), reinforcing immune escape pathways (119). Given the central role of MDSCs in ICI resistance, therapeutic strategies to deplete or inhibit these cells have gained interest. Colony-stimulating factor 1 receptor (CSF1R) inhibitors, such as pexidartinib, have shown promise in reducing MDSC populations and restoring T-cell function in AML (120). Similarly, small-molecule inhibitors of arginase-1, including INCB001158, are being explored to prevent MDSC-mediated depletion of L-arginine and restore T-cell proliferation. Phosphodiesterase-5 (PDE5) inhibitors, such as sildenafil, have also demonstrated efficacy in reducing MDSC-mediated immune suppression by modulating nitric oxide signaling (121).

Tregs are another key component of the AML tumor microenvironment that contribute to immune evasion and ICI resistance. These cells, characterized by high expression of the transcription factor FOXP3, suppress immune activation through multiple mechanisms, including cytokine secretion, direct inhibition of antigen-presenting cells, and metabolic competition. One of the primary pathways by which Tregs suppress immune responses is through the secretion of IL-10 and TGF-β, which inhibit the proliferation and function of effector T cells (122). Tregs also express high levels of cytotoxic T-lymphocyte-associated protein 4 (CTLA-4), which competes with the costimulatory molecule CD28 for binding to CD80/CD86 on antigen-presenting cells (APCs). This interaction reduces the activation of effector T cells and reinforces immune tolerance (123).

Therapeutic approaches to reduce Treg-mediated immune suppression have focused on selectively depleting these cells while sparing effector T cells. Monoclonal antibodies targeting CD25, a surface marker expressed on Tregs, such as basiliximab, have been tested to selectively deplete Tregs. Additionally, inhibitors of IDO, an enzyme that promotes Treg expansion by depleting tryptophan, such as epacadostat, are being investigated to block Treg-mediated immune suppression and enhance ICI responses. PI3K inhibitors, such as idelalisib, have also demonstrated the ability to reduce Treg populations and enhance antitumor immune responses (124).

Tregs contribute to AML immune escape by secreting immunosuppressive cytokines such as IL-10 and TGF-β. Indoleamine 2,3-dioxygenase (IDO) inhibitors, such as epacadostat, block Treg expansion and restore effector T-cell function, making them attractive candidates for combination therapy with ICIICIs (124). Additionally, pro-inflammatory cytokines such as IL-12 and IL-15 have been explored to enhance ICI efficacy in AML. IL-15 stimulates the proliferation of memory CD8+ T cells and natural killer (NK) cells, promoting long-term immune surveillance (125). IL-12 enhances CTL function by increasing IFN-γ production, thereby reversing myeloid immunosuppression (126).

### 4.4 Metabolic interventions

Metabolic reprogramming is a hallmark of cancer, allowing malignant cells to sustain rapid proliferation, evade immune surveillance, and resist therapy. In AML, tumor metabolism is intricately linked to immune evasion, and myeloid-derived suppressor cells (MDSCs) are a heterogeneous population of immature myeloid cells that accumulate in AML and potently suppress anti-leukemic immunity. Key metabolic adaptations in AML include increased reliance on glutamine metabolism, enhanced oxidative phosphorylation, and the depletion of essential amino acids such as tryptophan. These metabolic shifts not only support leukemia cell survival but also suppress immune function, contributing to checkpoint inhibitor (ICI) resistance.

Therapeutic strategies aimed at targeting these metabolic pathways offer a promising approach to reversing immune suppression and enhancing ICI efficacy. The major metabolic targets in AML immunotherapy include glutamine metabolism, the kynurenine pathway (mediated by indoleamine 2,3-dioxygenase [IDO]), and the tumor microenvironment’s (TME) hypoxic and nutrient-deprived conditions. MDSCs are expanded in AML patients compared to healthy controls and correlate with impaired T cell activation and reduced immunotherapeutic responsiveness. AML cells release factors and extracellular vesicles that drive MDSC expansion, providing a sustained source of these suppressive cells within the marrow niche. MDSCs suppress anti-leukemia immunity through expression of arginase-1 (Arg1), inducible nitric oxide synthase (iNOS), reactive oxygen species (ROS) generation, PD-L1 expression, and secretion of immunosuppressive cytokines such as TGF-β and IL-10. These mechanisms lead to T cell receptor downregulation, T cell anergy, and induction of regulatory T cells (Tregs), collectively undermining effective anti-tumor immunity (127).

Metabolic reprogramming fosters a suppressive metabolic microenvironment that acts in concert with checkpoint pathways to inhibit T cell function. Nutrient depletion by MDSCs limits T cell glycolysis and anabolic metabolism, which are prerequisites for effector differentiation and cytotoxicity. Concurrently, MDSC expression of PD-L1 and other inhibitory ligands engages PD-1 on T cells, reinforcing exhaustion programs. The intersection of metabolic suppression and checkpoint signaling thus constitutes a dual barrier to effective ICI therapy in AML (128). Metabolic cues also feedback on immune cell differentiation and function. These metabolic feedback loops, maintained by MDSCs and AML blasts, contribute to durable immunosuppression and therapeutic resistance (129).

Glutamine is an essential nutrient for AML cells, providing a critical source of nitrogen and carbon for nucleotide synthesis, redox balance, and bioenergetics. Unlike solid tumors, which frequently rely on glycolysis (the Warburg effect), AML cells exhibit high rates of oxidative phosphorylation (OXPHOS) and glutaminolysis to sustain their energy needs. Glutaminase, the enzyme responsible for converting glutamine to glutamate, plays a central role in AML metabolism. Inhibition of glutaminase has been shown to suppress AML growth and enhance immune responses. The glutaminase inhibitor CB-839 (telaglenastat) has demonstrated promising preclinical activity by disrupting AML metabolism, reducing tumor burden, and restoring T-cell function. CB-839 has been tested in clinical trials, both as monotherapy and in combination with checkpoint inhibitors, to determine its potential in overcoming ICI resistance (130). In addition to directly inhibiting leukemia growth, glutamine metabolism inhibitors can also enhance immune cell function. AML-driven glutamine consumption deprives T cells of this critical nutrient, leading to T-cell exhaustion and impaired effector function. By blocking glutamine metabolism in AML cells, these therapies may restore T-cell viability and improve the efficacy of ICIICIs.

Amino acid metabolism is another critical aspect of AML-driven immune suppression. The kynurenine pathway, regulated by the enzyme indoleamine 2,3-dioxygenase (IDO), plays a central role in this process by degrading tryptophan into immunosuppressive metabolites. AML cells and tumor-associated macrophages (TAMs) frequently overexpress IDO, leading to tryptophan depletion and accumulation of kynurenine, which inhibits effector T-cell activation and promotes regulatory T-cell (Treg) expansion. This metabolic shift results in an immunosuppressive TME that contributes to ICI resistance. IDO inhibitors, such as epacadostat, have been developed to block tryptophan metabolism and restore T-cell function. Preclinical studies have shown that IDO inhibition reverses T-cell exhaustion and enhances antitumor responses. Clinical trials evaluating IDO inhibitors in combination with PD-1/PD-L1 inhibitors have been conducted in solid tumors, but their role in AML remains under investigation (131).

Despite initial enthusiasm, single-agent IDO inhibition has yielded mixed results in clinical trials. However, combination approaches incorporating IDO inhibitors with ICIICIs, chemotherapy, or metabolic modulators may provide more durable responses. Further studies are needed to define optimal combinations and patient selection strategies. The bone marrow niche in AML is characterized by hypoxia and metabolic competition, both of which contribute to immune suppression and ICI resistance. Hypoxic conditions drive the stabilization of hypoxia-inducible factors (HIFs), which promote AML survival, induce immune checkpoint molecule expression, and suppress T-cell activity.

AML cells rely on mitochondrial metabolism and oxidative phosphorylation (OXPHOS) to sustain their growth and survival. In contrast to T cells, which require glycolysis for rapid activation and proliferation, AML cells depend heavily on mitochondrial respiration. This metabolic disparity creates an opportunity for therapeutic intervention. OXPHOS inhibitors, such as IACS-010759, selectively target mitochondrial metabolism in AML cells while preserving immune cell function. By blocking OXPHOS, these agents not only induce apoptosis in leukemia cells but also improve the metabolic fitness of T cells, enhancing their ability to respond to ICIICIs (132).

The excessive production of reactive oxygen species (ROS) is strongly elevated and correlates with the proliferation of AML cell lines (133). Furthermore, mitochondrial metabolism plays a crucial role in immune cell exhaustion. Dysfunctional mitochondria in T cells lead to increased ROS production, loss of mitochondrial membrane potential, and reduced ATP generation. Strategies to enhance mitochondrial function, such as PGC-1α activation or mitochondrial transfer from stromal cells, have been explored to reinvigorate exhausted T cells and improve ICI responses.

In AML, metabolic suppression orchestrated by MDSCs constitutes a major mechanism of immune evasion and resistance to ICI therapy. MDSCs exploit metabolic pathways—including glucose metabolism, amino acid catabolism, and fatty acid oxidation—to sustain their suppressive functions and deplete essential nutrients from the TME. This metabolic reprogramming synergizes with immune checkpoints such as PD-1/PD-L1 to create a profoundly immunosuppressive niche that hampers effective antitumor responses. Targeting these metabolic circuits in conjunction with checkpoint blockade represents a compelling therapeutic strategy to enhance immunotherapy outcomes in AML. While metabolic interventions hold promise for overcoming ICI resistance in AML, several challenges remain. The metabolic landscape of AML is highly heterogeneous, with distinct subpopulations of leukemia cells exhibiting different metabolic dependencies. Identifying metabolic vulnerabilities specific to ICI-resistant AML cells will be critical for developing effective targeted therapies. Additionally, systemic metabolic interventions may have unintended consequences on immune cells. For example, while glutaminase inhibitors can suppress AML growth, excessive inhibition of glutamine metabolism may impair T-cell function. Future research should focus on developing selective metabolic modulators that preferentially target AML cells while preserving immune responses. Combination strategies integrating metabolic inhibitors with ICIICIs, chemotherapy, and immune-modulating agents may provide the most effective approach to overcoming resistance. Ongoin clinical trials are evaluating the safety and efficacy of metabolic inhibitors in AML, and their results will provide valuable insights into the role of metabolism in ICI resistance.

### 4.5 Personalized approaches and biomarker-driven therapy

Ultimately, a personalized approach to checkpoint inhibitor therapy is essential for optimizing treatment responses. Genomic and transcriptomic profiling can help potentially identify biomarkers predictive of response, enabling patient stratification. There has been investigation into various models to potentially predict response to immunotherapy in solid tumor applications. One such fitness model focused on immune interaction of neoantigens based on the likelihood of neoantigen presentation by the major histocompatibility complex and subsequent recognition by T cells, and demonstrated applicability in predicting survival of CTLA-4 treated patients with melanoma and PF-1 treated patients with lung cancer (134). Integrating these approaches into clinical practice may improve patient outcomes and reduce unnecessary exposure to ineffective treatments.

Conventional biomarkers such as PD-L1 expression and tumor mutational burden (TMB) are insufficient predictors of response ot ICIs in many hematologic contexts, motivating the adoption of integrative, multi-omic approaches that combine layers of molecular data to better forecast therapeutic outcomes. Multi-omic platforms hold significant promise for improving the prediction of ICI responses in hematologic malignancies by capturing the complex interplay between tumor cells and the immune system. Recent studies collectively indicate that prediction of ICI response in AML requires integrative multi-omic approaches that capture immune state, tumor biology, and microenvironmental context. Transcriptomic and single-cell profiling currently provide the strongest predictive signals, while genomics, proteomics, metabolomics, and liquid biopsy approaches offer complementary insights. Continued refinement of integrative models and validation in prospective AML immunotherapy trials will be essential to enable precision immunotherapy in this challenging disease (135–138).

Current classification systems for AML (ELN 2022) primarily focus on cytogenetic and somatic mutations in leukemic blasts. Presence of prognostic (e.g. *TP53, MECOM*) or targetable *(e.g. IDH, FLT3*) mutations determine prognosis is defined by overall survival and decision to offer allogeneic stem cell transplantation to patient or not. The role of immune microenvironment (IME) has been less studied in determining eventual prognosis of these patients and how this may correlate with response to ICI therapies. In solid tumors response to ICI therapy is correlated with infiltration of CD8 positive T cells, expression of inflammatory cytokines and high T-cell receptor clonal diversity pretreatment. This inflamed microenvironment correlates with high expression of IFN-γ responsive genes that may overcome immune escape conferred by IDO mutations and down-regulation of PDL1 in cancer cells. However, overexpression of IFN-γ signaling activates STAT1 mediated resistance to ICI therapy in preclinical models. Vadakekolathu et al. derived immune signatures based on mRNA expression from bone marrow biopsy specimens of treatment naïve AML patients. Based on this, patients were classified into three subgroups which included an IFN-γ dominant group, adaptive immune response group, and myeloid cells abundance groups (139). The subgroups subsequently dichotomized into immune infiltrated and immune depleted subgroups based on gene expression in the tumor microenvironment. In the immune infiltrated subgroup, patients demonstrated expression of IFN-γ stimulating genes and T-cell recruiting genes. In patients who were classified into favorable risk by ELN criteria presence of high immune infiltration gene signature was associated with improved OS, whereas the converse was noted in patients with ELN adverse risk AML.

Similar results were noted by Lasry et al. where inflammation associated gene score (iScore) correlated with clinical outcomes in ELN favorable and adverse risk AML. This score was derived by incorporating bone marrow infiltration with specific immune cells including CD8+GZMK, Tregs and atypical B cells (140, 141). Data from large databases such as TGCA, MDACC and BEAT -AML also showed that IFN-γ signaling score correlates with worse survival (22). Moreover, in monocytic AML (M4, M5) IFN-γ producing T and NK cells promotes chemotherapy and venetoclax resistance. Similarly, CD4 T cells with Th1 signature (high TNFa IFNg) predicts chemoresistance and poor OS, but conversely is predictive of response to ICI (142). With greater recognition of the role of microenvironment, and the influence of gene expression patterns leading to inflamed signature in TME, future clinical trials can explore incorporation of ICI therapies in patients who are predicted to respond based on these findings.

## 5 Future directions and challenges

Despite advances in understanding checkpoint inhibitor resistance, several challenges remain. Optimizing patient selection criteria, mitigating immune-related toxicities, and identifying reliable biomarkers for response prediction are key areas of ongoing research. Novel immune checkpoint targets and rational combination strategies need further validation in preclinical and clinical settings. Future efforts should focus on integrating personalized immunotherapeutic approaches to enhance checkpoint inhibitor efficacy in AML. Ongoing clinical trials are investigating these various strategies, but clinical translation remains a challenge due to the complexity of AML biology and the diversity of the immune landscape in different patients. Optimizing the sequencing and timing of therapies, along with identifying predictive biomarkers, will be key to advancing this field. As the understanding of AML’s immune resistance mechanisms improves, it is likely that combination approaches, personalized treatment strategies, and the development of novel immune-modulatory agents will pave the way for more effective therapies in the future.

## 6 Conclusion

Checkpoint inhibitor resistance in AML presents a formidable challenge, but emerging strategies offer hope for improved outcomes. The mechanisms of resistance are complex and multifactorial, involving both tumor-intrinsic factors and immune-related processes. By understanding and targeting these resistance pathways, researchers are developing novel strategies to enhance the effectiveness of checkpoint blockade therapies (**Figure 1**). Combination therapies, modulation of the tumor microenvironment, T-cell enhancement strategies, and genetic and epigenetic approaches offer exciting opportunities to overcome resistance and improve outcomes for patients with AML. While challenges remain, ongoing research into the immunobiology of AML and the optimization of therapeutic strategies holds the potential to significantly improve the prognosis for patients with this aggressive disease. A deeper understanding of resistance mechanisms and innovative therapeutic combinations will be essential to fully harness the potential of immunotherapy in AML. Continued research efforts and clinical trials will be crucial in establishing checkpoint inhibitors as a viable treatment modality for AML.
